# The Effects of Whey vs. Pea Protein on Physical Adaptations Following 8-Weeks of High-Intensity Functional Training (HIFT): A Pilot Study

**DOI:** 10.3390/sports7010012

**Published:** 2019-01-04

**Authors:** Amy Banaszek, Jeremy R. Townsend, David Bender, William C. Vantrease, Autumn C. Marshall, Kent D. Johnson

**Affiliations:** Exercise and Nutrition Science Graduate Program, Lipscomb University, Nashville, TN 37204, USA; Amy.banaszek@gmail.com (A.B.); david.bender85@gmail.com (D.B.); vantreaswc@lipscomb.edu (W.C.V.); autumn.marshall@lipscomb.edu (A.C.M.); kent.johnson@lipscomb.edu (K.D.J.)

**Keywords:** crossfit, exercise, amino acids, strength

## Abstract

This study examined the effects of whey and pea protein supplementation on physiological adaptations following 8-weeks of high-intensity functional training (HIFT). Fifteen HIFT men (n = 8; 38.6 ± 12.7 y, 1.8 ± 0.1 m, 87.7 ± 15.8 kg) and women (n = 7; 38.9 ± 10.9 y, 1.7 ± 0.10 m, 73.3 ± 10.5 kg) participated in this study. Participants completed an 8-week HIFT program consisting of 4 training sessions per week. Participants consumed 24 g of either whey (n = 8) or pea (n = 7) protein before and after exercise on training days, and in-between meals on non-training days. Before and after training, participants underwent ultrasonography muscle thickness measurement, bioelectrical impedance analysis (BIA), two benchmark WODs (workout of the day), 1-Repetition Maximum (1RM) squat and deadlift testing, and Isometric Mid-thigh Pull (IMTP) performance. Separate analyses of covariance (ANCOVA) were performed on all measures collected at POST. Both groups experienced increased strength for 1RM back squat (*p* = 0.006) and deadlift (*p* = 0.008). No training effect (*p* > 0.05) was found for body composition, muscle thickness, IMTP peak force, IMTP rate of force development, or performance in either WOD. Using PRE values as the covariate, there were no group differences for any measured variable. We conclude that ingestion of whey and pea protein produce similar outcomes in measurements of body composition, muscle thickness, force production, WOD performance and strength following 8-weeks of HIFT.

## 1. Introduction

It is well established in the literature that individuals engaging in elevated physical activity have greater protein needs [1,2,3]. Current literature suggests 1.4–2.0 g/kg/d is sufficient to support performance adaptations, improve body composition, and sustain accretion of lean body mass while engaged in a resistance training regimen [1]. While meeting daily protein macronutrient goals is of primary importance to athletes, the quality of protein consumed also has been shown to impact resistance training adaptation [4]. Protein quality is generally determined by the ability of a protein to meet the demand for amino acids while considering its digestibility and utilization by the body [5]. In the context of resistance training, a high-quality protein is one which possesses all the essential amino acids (EAA) and contains a sufficient amount of the branched chain amino acid (BCAA) leucine to augment muscle protein synthesis (MPS).

Due to its high leucine content, rapid digestibility, and ability to maximally stimulate MPS, whey protein is a common choice for protein supplementation among active and athletic populations [6,7]. Whey protein supplementation in conjunction with resistance training has been shown to promote larger gains in muscle mass and strength compared with a non-energetic placebo [8,9] and isocaloric carbohydrate supplements [10,11,12]. While whey protein supplementation appears to enhance adaptations to resistance training, not all athletes are able or willing to consume whey or animal proteins [13,14]. Athletes adhering to a plant-based diet or those who present with other dietary restrictions often turn to soy or other plant proteins as a comparable substitute for whey [13]. While the bulk of the literature comparing outcomes of whey and soy protein supplementation in conjunction with resistance training have been equivocal [15,16,17,18], a recent investigation demonstrated that 9-months of resistance training produced significantly larger gains in lean body mass in a whey supplemented group compared with soy [19]. Based on these findings and others which seem to displace soy as viable substitute for whey [6,17,19,20], there is continued interest in other protein sources which show potential as a comparable alternative to whey. Recently, Babault et al., [21] reported that pea protein supplementation produced similar increases in muscular size and strength in comparison to a whey supplement following elbow flexor and extensor resistance training. While these results appear promising for those adhering to plant-based diets, this study did not utilize the full-body dynamic resistance training that is representative of most athletic training. As such, findings from this investigation may be limited in their practical application to individuals engaged in rigorous training.

High-Intensity Functional Training (HIFT) is a relatively new training mode which has recently emerged in the fitness industry. The interest in HIFT may be due in large part to a strong camaraderie among its participants, stimulating increased program adherence compared to traditional resistance training [22,23,24]. Physiologically, contemporary research suggests that HIFT may provide improvements in body composition (10, 17) and increase aerobic capacity (17, 18), while also increasing relative and absolute strength (10). The salient feature of HIFT is the inclusion of high-intensity dynamic resistance training coupled with endurance-related repetition schemes with little or no rest in-between exercises [25]. As a result, high caloric expenditures have been reported which mimic caloric outputs of much longer resistance circuit-style training (23). Despite its popularity, surprisingly few studies have detailed training adaptations following chronic HIFT and even less is known regarding dietary guidelines to optimize HIFT. Furthermore, some subgroups of HIFT communities adhere to firm dietary practices, some of which require the removal of certain high-quality protein sources (e.g., dairy products) from one’s diet [26]. Subsequently, HIFT athletes commonly turn to other plant-based protein sources (e.g., pea, rice, soy) to meet their macronutrient goals and support training adaptations. While recent developments in HIFT literature have provided an initial glimpse into the social and physiological implications of this training style, additional work is needed to further describe expected HIFT adaptations and provide evidence for appropriate HIFT dietary guidelines.

Therefore, the purpose of the present pilot study was to compare the effects of whey and pea protein supplementation in conjunction with 8-weeks of HIFT on strength, body composition, muscle thickness, IMTP peak force, IMTP RFD, and WOD performance. Based on previous findings, we hypothesized that similar adaptations to HIFT would occur with whey and pea protein treatments. Additionally, we hypothesized that HIFT would produce improvements maximal strength, sport-specific performance, muscle thickness, isometric force production and body composition in HIFT trained participants.

## 2. Materials and Methods

### 2.1. Participants

Fifteen HIFT trained men (n = 8, 38.6 ± 12.7 y, 1.8 ± 0.1 m, 87.7 ± 15.8 kg) and women (n = 7, 38.9 ± 10.9 y, 1.7 ± 0.10 m, 73.3 ± 10.5 kg) volunteered to participate in this randomized, double-blind training study. All participants had been participating in at least three HIFT workouts per week for at least six months and were free of any physical limitations that may affect performance. Additionally, all participants were free of any medications and performance enhancing drugs, as determined and confirmed by a health and activity questionnaire and all participants agreed to abstain from dietary supplements (e.g., creatine, beta-alanine, amino acids, pre-workout) throughout the duration of their enrollment in the study. Habitual caffeine (e.g., coffee, tea) users were allowed to continue use outside of peri-workout nutrition. Following an explanation of all investigative procedures, potential risks, and possible benefits, each participant provided their informed consent prior to involvement in this study. The research protocol was approved by the University’s Institutional Review Board prior to any participant enrollment.

### 2.2. Study Protocol

Following a familiarization session, participants were tested in the Human Performance Lab for baseline body composition, muscle thickness, and isometric force measurements. To eliminate the potential for dietary influence on muscle architecture and body composition, participants reported to the lab for baseline testing on a 10-h overnight fast. Moreover, participants were instructed to report to the lab and for all performance testing hydrated while abstaining from caffeine, alcohol, and vigorous exercise for at least 24 h prior to both lab testing sessions. Activity more intensive than light running, stretching, or calisthenics was defined as vigorous (e.g., anaerobic conditioning, sprinting, or resistance training). When participants reported for testing, these conditions were confirmed verbally by an investigative team member. Additional performance testing occurred at a local CrossFit gym where all participants completed 1-repetition maximal strength (1RM) testing on the back squat and deadlift exercises and two Workout of the Day (WOD) performance bouts.

### 2.3. Body Composition Testing

Multi-frequency Bioelectrical Impedance Analysis (BIA) was completed at the beginning of and directly after the 8-week intervention using InBody^®^ 570 Body Composition Analyzer (Biospace, Inc., Seoul, Korea). Body composition information was gathered in this manner by measuring the body’s reactance and resistance to microamperes of electrical current, and differing frequencies are used to distinguish between tissue and water mass and placement. There are 8 total electrodes, located on each thumb, hand, sole of the foot, and pad of the foot. Participants were instructed to wipe the bottom of their feet with an antibacterial wipe containing electrolytes to aid in current movement through the feet. Weight was measured by a scale within the device, after which age, height, and gender are manually entered. The participant was then instructed to stand erect, with arms away from the body and stretched out straight to their sides, while refraining from moving or talking. Body fat percentage for the entire body was automatically calculated utilizing body mass and impedance via equations supplied in the manufacturer’s proprietary software. 

### 2.4. Isometric Mid-Thigh Pull Testing

Each participant’s mid-thigh position was determined before testing by marking the midpoint distance between the knee and hip joints. Participants assumed their preferred second pull power-clean position by self-selecting their hip and knee angles. The height of the barbell was adjusted up or down (±1.0 in) to make sure it was in contact with the mid-thigh. All participants used an overhand grip and participants were strapped to the barbell similar to previous investigations [27,28]. All participants were instructed to relax before the command “GO!” to avoid pre-contraction. The researchers instructed participants to pull upwards on the barbell as hard and as fast as possible and to continue their maximal effort for 6 s, in a manner similar to standard clean movements. The force-time curve for each trial was recorded by dual force plates (PASCO, Roseville, CA, USA) with a sample rate of 1000 Hz similarly to previous investigations [27,28].

Peak force was defined as the highest force achieved during the 6-s isometric test minus the participant’s body weight in Newtons. The rate of force development (RFD) was then calculated with the following equation: RFD = ΔForce/ΔTime. The RFD equation was applied to a predetermined time band (0–250 ms). This was in accordance with previous work which used a similar predetermined time band when RFD demonstrating high reliability [27].

### 2.5. Muscle Ultrasound Measurement

Muscle thickness (MT) was captured using B-mode ultrasound imaging with a 12 MHz linear probe coated in a water-based conduction gel (General Electric LOGIQ P5, Wauwatosa, WI, USA). Prior to muscle image capture, participants laid supine for 5 min. For measurements of MT, the probe was oriented longitudinally in the sagittal plane parallel to the muscle tissue without depressing the skin. Image captures for the rectus femoris (RF) were taken at 50% of the distance from the anterior, inferior suprailiac spine to the most proximal point of the patella [29]. Vastus lateralis (VL) images were obtained by the same means as previously stated; however, the sampling location is determined by 50% the straight-line distance between the greater trochanter and the lateral epicondyle of the femur [29]. Following image collection, MT analysis was completed using Image J software on a separate lab computer (version 1.45 s; National Institutes of Health, Bethesda, MD, USA). MT was derived from the still muscle image as the distance between the inferior border of the superficial aponeurosis and the superior border of the deep aponeurosis. Intraclass correlation coefficient (ICC_3,k_), standard error of measurement (SEM), and minimal difference (MD) measures for the single ultrasound technician were calculated for the RF MT (ICC_3,k_ = 0.99, SEM_3,k_ = 0.05, MD = 0.15 cm) and VL MT (ICC_3,k_ = 0.97, SEM_3,k_ = 0.08, MD = 0.22 cm) from analysis of 10 individuals separated by 24 h.

### 2.6. Maximal Strength Testing

Prior to and directly following the 8-week study, participants reported to a local CrossFit gym (CrossFit West Nashville, Nashville, TN, USA) to establish their 1RM for the straight bar back squat and deadlift exercises. Immediately before 1RM assessment, all participants performed an identical warm-up consisting of 10 body weight squats, 10 body weight walking lunges, 10 dynamic walking hamstring stretches, and 10 dynamic walking quadriceps stretches. The 1RM test for the barbell back squat and deadlift were performed using the methods previously described [28]. Briefly, each participant performed two warm-up sets using a resistance of approximately 40–60% and 60–80% of their perceived maximum, respectively. For each exercise, 4–5 subsequent attempts were performed to determine the 1RM. At least 3 min of rest was provided between each 1RM attempt. Any 1RM attempt visually not meeting the range of motion criteria for each exercise or where proper technique was not maintained was not considered a successful lift and was discarded. 

### 2.7. Workout of the Day (WOD) Testing

Prior to and directly after the 8-week training program, participants completed two separate WODs (WOD1, WOD2). Immediately before WOD performance, a group warm-up was led by a trainer consisting of running, bodyweight squats, walking lunges, stretches, and box jumps. WOD1 required the participants to complete the following as fast as possible, in this order: 400 m run, 50 sit ups, 400 m run, 40 box jumps (24″ box for men, 20″ for women), 400 m run, 30 kettle bell swings (24.1 kg for men, 15.9 kg for women), 400 m run, 20 wall balls (9.1 kg to a 10′ target for men, 6.4 kg to a 9′ target for women), 400 m run, and 10 thrusters (43.2 kg for men, 29.5 kg for women). For WOD1, time to completion (s) was recorded by study personnel and used for analysis. Following a 5-min rest period, participants completed WOD2, which consisted of a maximum distance row using a row ergometer (Concept 2^®^, Rogue, Columbus, OH, USA) in 2 min. Upon completion, a participants’ score was recorded (meters rowed) and used for further analysis.

### 2.8. Supplementation Protocol

Participants were matched based on sex and squat 1RM and were randomly assigned to either the whey (men = 4, women = 3) or pea protein (men = 4, women = 4) group. Participants were provided individual bags with their assigned study supplement and consumed their respective supplement twice daily for the entire 8-week study. Each bag had one serving of whey protein (WP: 120 kcal, 1.2 g fat, 1.4 g CHO, 24.4 g PRO) or pea protein (PP: 110 calories, 1.2 g fat, 1 g CHO, 24.5 g PRO) purchased from a supplement provider (True Nutrition, Vista, CA, USA). The amino acid profiles of both supplements are provided in Table 1. For those who habitually consumed protein supplements, participants halted consumption of their normal protein supplement and replaced it with the protein supplied by the researchers. Participants mixed protein with ~350 mL of water in supplied shaker bottles, and consumption occurred within an hour before and after the training session on workout days. On non-training days, participants consumed their respective supplement once in the morning and once in the evening in between meals. Participants were required to return their used supplement bags to a member of research team at the end of every week to ensure supplement compliance. At this time, the supplement for the following week was provided to each participant.

### 2.9. Dietary Logs

During the HIFT training period and supplement intervention participants were asked to record daily food logs throughout the entire study. Investigators collected a digital record of participants’ daily food logs every week when supplement bags were issued to participants. Dietary recalls were used to provide an estimate of total kilocalorie intake (kcal) and macronutrient distributions (carbohydrate, protein, and fat) of the participant’s daily diet. All participants recorded their dietary consumption utilizing the MyFitnessPal application (Under Armour Inc., Baltimore, MA, USA). This application service contains a large, detailed US-branded food catalogue and has been validated against paper-based written food logs [30]. 

### 2.10. 8-Week High Intensity Functional Training Intervention

Throughout the 8-week intervention, every HIFT workout was led by a CrossFit certified trainer to ensure proper form and adherence to the programmed intensity and volume of the workout. The WOD programmed for any particular day is the same WOD completed by all members at the gym. All training sessions consisted of a strength training session, followed by a metabolic conditioning session. Strength training sessions employed progressive overload and included intensities ranging from 60–100% of a participants 1RM for a given exercise. Metabolic conditioning sessions included a variety of common Olympic and strength movements (e.g., cleans, snatches), bodyweight exercises (e.g., Air Squats, push-ups, pull-ups), non-traditional training methods (e.g., kettlebell swings, medicine ball throws) and other anaerobic activities (e.g., jump rope, row ergometer, sprinting). Overall, regular movements included in these CrossFit workouts included push-ups, pull-ups, burpees, variations of cleans and snatches, deadlifts, squats, running, rowing on a cycle ergometer, wall balls, kettle bell swings, thrusters, and box jumps. Additionally, in order for all participants to have the ability to participate and complete a workout, scaled options were provided by a trainer if an individual was unable to complete a more complex task (e.g., box jump, handstand push-ups, pull-ups) or higher intensity. These scaled exercises utilized similar musculature as the prescribed exercise while lowering the intensity or skill-components for a novice participant to complete the workout. If a participant was not able to complete a workout at their assigned day and time, they were allowed to make up the prescribed workout at the gym at a more convenient time. To ensure compliance and completion of all workouts, participants were required to record their workout performance in a software program provided by the gym (Wodify, Cherry Hill, NJ, USA). 

### 2.11. Statistical Analysis

Prior to hypothesis testing, the Shapiro-Wilk test was used to evaluate the assumption of normality for dependent variables. To detect differences between the experimental treatments on changes in 1RM strength, IMTP force, WOD performance, body composition, and muscle thickness an analysis of covariance (ANCOVA) was performed on all variables collected at POST. Associated values obtained at PRE were used as the covariate to eliminate the possible influence of initial score variances on the outcomes. Following a significant F-ratio, a paired-samples t-test was used to determine if significant differences existed between measures collected prior to and immediately following 8 weeks of HIFT. Group differences were further assessed via effect sizes (η^2^p; partial eta squared). Effect sizes were interpreted as small (0.01–0.059), medium (0.06–0.139), or large (>0.14) as previously recommended [31]. An independent t-test was used to assess differences in average caloric and macronutrient intake between groups over the 8-week intervention. An alpha level was set at *p* ≤ 0.05, and all analyses were performed using SPSS version 24.0 (SPSS, Inc., Chicago, IL, USA).

## 3. Results

All dietary recalls and empty supplement bags were returned each week and participants reported full compliance with no reported adverse events. Dietary analysis of the 8-week intervention revealed no significant differences between average calorie intake (*p* = 0.302; WP: 2287.8 ± 704.8 kcals; PP: 1888.1 ± 295.8 kcals), protein intake (*p* = 0.619; WP: 150.7 ± 40.9 g; PP: 129.9 ± 32.1 g), carbohydrate intake (*p* = 0.169; WP: 191.6 ± 60.4 g; PP: 165.8 ± 39.1 g), or fat intake (*p* = 0.284; WP: 88.7 ± 29.6 g; PP: 66.52 ± 17.7 g) throughout the course of the investigation. Furthermore, average protein consumption relative to body weight was not significantly different between groups (*p* = 0.427; WP: 1.8 ± 0.3 g/kg; PP: 1.7 ± 0.4 g/kg).

### 3.1. Strength, IMTP, and WOD Performance

Whey and pea supplement groups both experienced significant improvements in maximal strength as a result of the resistance training program (Table 2). Significant improvements for the 1RM squat (*p* = 0.006) and 1RM deadlift (*p* = 0.008) were found, but no significant differences in 1RM improvements were observed between whey and pea groups. No significant improvements in IMTP peak force (*p* = 0.991) or IMTP RFD at 250 ms (*p* = 0.997) were observed as a result of the training intervention, with no significant differences between groups. There were no significant improvements found for WOD1 (*p* = 0.157) or WOD2 (*p* = 0.07) as a result of 8-weeks of HIFT training with no differences found between whey and pea protein conditions.

### 3.2. Body Composition & Muscle Thickness

There was no significant main effect for time for body mass (*p* = 0.100) or body fat percentage (*p* = 0.336) following the 8-week intervention with no differences found between groups (Table 3). Rectus femoris (*p* = 0.593) and vastus lateralis (*p* = 0.678) muscle thickness remained unchanged with no differences found between groups. 

## 4. Discussion

The main purpose of this pilot study was to investigate the effects of pea and whey protein supplementation on HIFT outcomes. Additionally, we sought to further examine training adaptations which occur as a result of chronic HIFT. Our findings showed no significant difference in type of protein consumption on strength, body composition, muscle thickness, IMTP peak force, IMTP RFD, and WOD adaptations following HIFT. Furthermore, 8 weeks of HIFT training resulted in significant improvements in muscular strength for the back squat and deadlift for both groups. However, HIFT did not result in improvements in body composition, muscle thickness, IMTP or WOD performance.

In the present study, both groups experienced significant increases in 1RM squat and deadlift strength which supports previous reports of HIFT induced improvements in strength [32]. No differences in strength were observed between whey and pea protein groups. In support of these data, training studies do not always demonstrate that protein quality correlates with improved chronic strength and performance outcomes [17,33,34]. Hartman et al., [17] found significant increases in strength for whey, soy, and casein protein groups without differences between groups following 12-weeks of resistance training. Joy et al., [34] found that providing 48 g doses of whey and rice protein, theoretically maximizing MPS with each dose, lead to similar increases in 1RM bench press and leg press, and increases in peak power in a Wingate test. To date, only one study has investigated the effect of pea protein supplementation accompanying a resistance training routine. Babault et al., [21] found a significant increase in 1RM and muscle torque for upper-body maximal voluntary torque during isometric, concentric, and eccentric elbow flexions, as well as 1RM bicep curl, without a significant difference between pea and whey protein supplements. The present study was designed implementing supplements with closely matched leucine content (WP: 2.2 g/dose, PP: 2.1 g/dose). Regardless of the potential digestibility differences between supplements, leucine similarities may explain why we saw no differences between groups for training outcomes [7]. This was demonstrated in an investigation where the addition of BCAAs to a soy supplement further augmented strength gains in elderly patients above that of a soy supplement alone [35]. As data regarding the effects of pea protein ingestion on the plasma amino acid response and mixed muscle protein synthesis is non-existent, more research is needed to understand if digestion alters pea protein amino acid availability and transport compared to whey.

We did not see any significant changes in body composition measurements of any kind following HIFT training regardless of supplemental condition. Moreover, we saw no significant increase in muscle size in either groups as a result of 8-weeks of HIFT. Likewise, in trained military personnel, 8-weeks of HIFT also did not produce significant improvements in body composition [36]. Similarly, no improvements in fat mass, lean body mass, or muscle thickness were found following 12-weeks of HIFT training in CrossFit trained men and women [37]. In the longest HIFT study to date, Feito et al. [32] showed that 16-weeks of HIFT resulted in significant improvements in body fat percentage, lean body mass, and elevations in bone mineral content. Therefore, given that our participants had already been participating in HIFT for at least 6 months prior to enrollment, it is likely that our 8-week intervention was not sufficient in duration to see significant alterations in body composition, or detect changes between groups. A strength of our study was the daily tracking of caloric intake. While daily protein (1.7 g/kg/d) intake was sufficient to support muscle adaptation, our participant’s overall caloric intake (~2088 kcals/day) may not have been sufficient to support large increases in overall weight, lean body mass, or muscle size [2,18]. It is also important to note that while our participants consumed either a plant or animal supplement before and after each workout, none of our participants adhered to a vegan or vegetarian lifestyle. As such, future investigations involving vegan or vegetarian athletes which consume pea protein as their primary protein source would be valuable in assessing its effectiveness compared to a diet of mostly animal proteins. 

Although we saw increases in 1RM strength for both groups, we did not see any improvements in IMTP peak force or IMTP RFD. This was somewhat surprising due to the emphasis on the inclusion of Olympic lifts in this HIFT program. This phenomenon has also been observed with traditional resistance training, as peak RFD was shown to have a weak relationship with 1RM values in trained college athletes [38] and recreational lifters [39]. While familiarization was provided, the lack of improvement in IMTP performance may have resulted from participant inexperience with the IMTP test. Additionally, although Olympic movements are regularly implemented as a part of HIFT, these exercises are not always practiced at intensities corresponding with the development of high force outputs [40,41]. HIFT is most commonly implemented and designed to promote all-around fitness, utilizing low-to-moderate intensity, high volume training. As has previously been demonstrated, high-intensity resistance training produces greater improvements in peak isometric force compared to a moderate intensity, high volume program [42]. Therefore, it’s not surprising that RFD and peak force did not increase as workouts were not designed specifically to promote peak force outputs. 

A curious finding was that no improvements in WOD performance, regardless of supplement group, were found following our HIFT intervention. Feito et al. [32] reported improvements in three separate WOD workouts after 16-weeks of HIFT. Yet, minimal improvements in WOD [41] and anaerobic performance [37] have been reported following shorter HIFT (6 and 12 weeks respectively) programs. In the present study, one limitation was that our WOD workouts were not counterbalanced as WOD2 took place after WOD1 and was likely affected by the physical demands of WOD1. In future work, counterbalancing WOD workouts would allow for more accurate assessment. Additionally, the WODs chosen for the present study were not considered “benchmark WODs”, or WODs commonly completed in CrossFit gyms worldwide. As such, utilizing benchmark WODs in our testing may improve the sensitivity of our assessments in future studies. Recent work by Mangine et al., [43] has provided normative values for benchmark workout which will aid in future investigations which seek to characterize the anaerobic performance adaptations associated with HIFT.

## 5. Conclusions

To summarize, our data suggest that whey and pea proteins promote similar strength, performance, body composition, and muscular adaptations following 8-weeks of HIFT. A clear limitation to our study was the limited number of participants. As such, these data should be interpreted within this framework. To our knowledge, this is just the second study to investigate the effects of pea protein supplementation in conjunction with chronic resistance training. Our findings also demonstrated that 8 weeks of HIFT resulted in significant improvements in muscular strength for the back squat and deadlift for both groups with no other detected improvements. Another limitation to our study was that no control group which did not engage in HIFT was enrolled. Subsequent work is needed utilizing a non-exercising control or a traditional resistance training group to distinguish if HIFT provided salient benefits compared to chronic traditional resistance training. While limited in participant numbers, this study provides additional evidence regarding suitable alternatives to whey protein for athletes with dietary restrictions. Additionally, it contributes to our further understanding of HIFT and accompanying adaptations from participation in this rapidly growing training mode. 

## Figures and Tables

**Table 1:** Amino acid composition in g/100 g of whey and pea protein supplements.

Amino Acid	Whey Protein	Pea Protein
Alanine	3.5	4.3
Arginine	2.3	8.7
Aspartic Acid	8.4	11.5
Cystine	1.7	1
Glutamic Acid	13.3	16.8
Glycine	1.4	4.1
Histidine	1.6	2.5
Isoleucine	4.6	4.5
Leucine	8.8	8.4
Lysine	7.5	7.2
Methionine	1.6	1.1
Phenylalanine	2.6	5.5
Proline	6.6	4.5
Serine	4.6	5.3
Threonine	4.5	3.9
Tryptophan	1.3	1
Tyrosine	2.3	3.8
Valine	4.4	5

**Table 2:** Performance changes following 8-weeks of high-intensity functional training.

Variable	Treatment	PRE	Covariate	POST	F	*p*	η^2^	95% Confidence Interval	
								Lower	Upper
Squat 1RM (kg)	Whey	107.7 ± 27.5	102.6 ± 35.9	111.7 ± 25.0	2.323	0.153	0.162	100.9	110.5
	Pea	98.6 ± 43.5		104.8 ± 42.5				105.6	115.9
Deadlift 1RM (kg)	Whey	135.4 ± 32.8	132.1 ± 37.5	142.5 ± 30.8	0.016	0.903	0.001	132.2	144.7
	Pea	129.3 ± 43.3		134.4 ± 46.7				131.2	144.6
IMTP PF (N)	Whey	2420.8 ± 207.9	2374.5 ± 521.0	2419.9 ± 412.3	0.741	0.408	0.063	2113.1	2522.5
	Pea	2339.8 ± 188.7		2341.8 ± 211.6				2228.0	2637.4
IMTP RFD 250 ms (N)	Whey	5497.4 ± 911.9	5605.0 ± 2170.4	5689.1 ± 2019.6	0.020	0.892	0.002	4000.9	7433.6
	Pea	5685.6 ± 756.1		5594.2 ± 1622.4				3981.7	7158.4
WOD 1 (s)	Whey	855.1 ± 62.8	870.1 ± 48.8	857.0 ± 63.0	3.572	0.083	0.229	846.8	898.0
	Pea	883.1 ± 31.1		856.1 ± 45.4				810.9	865.9
WOD 2 (m)	Whey	529.2 ± 74.3	560.3 ± 134.0	515.3 ± 66.7	0.008	0.931	0.001	526.9	569.7
	Pea	528.6 ± 48.6		517.1 ± 40.2				526.7	571.9

Data presented as mean ± SD. IMTP = Isometric Mid-thigh Pull. RFD = Rate of Force Development. WOD = Workout of the Day.

**Table 3:** Changes in body composition following 8-weeks of high-intensity functional training.

Variable	Treatment	PRE	Covariate	POST	F	*p*	η^2^	95% Confidence Interval	
								Lower	Upper
Body Mass (kg)	Whey	83.9 ± 18.9	81.0 ± 15.1	83.7 ± 18.5	0.104	0.752	0.009	79.7	81.5
	Pea	78.4 ± 11.6		77.8 ± 11.6				79.5	81.3
Body Fat (%)	Whey	22.0 ± 7.7	22.1 ± 6.78	22.1 ± 7.3	0.220	0.647	0.018	21.2	22.7
	Pea	22.3 ± 6.4		21.6 ± 6.6				20.9	22.5
Rectus Femoris Thickness (cm)	Whey	2.51 ± 0.51	2.36 ± 0.48	2.50 ± 0.47	0.107	0.750	0.009	2.23	2.60
	Pea	2.24 ± 0.44		2.31 ± 0.39				2.18	2.57
Vastus Lateralis Thickness (cm)	Whey	1.80 ± 0.43	1.59 ± 0.50	1.74 ± 0.48	0.622	0.447	0.054	1.46	1.89
	Pea	1.49 ± 0.55		1.45 ± 0.24				1.31	1.81

Data presented as mean ± SD.
